# Genome-Wide Identification and Expression of MAPK Gene Family in Cultivated Strawberry and Their Involvement in Fruit Developing and Ripening

**DOI:** 10.3390/ijms23095201

**Published:** 2022-05-06

**Authors:** Mengyao Li, Binghua Li, Min Yang, Liangxin Wang, Guoyan Hou, Yuanxiu Lin, Yunting Zhang, Yong Zhang, Qing Chen, Yan Wang, Wen He, Xiaorong Wang, Haoru Tang, Guichuan Yang, Ya Luo

**Affiliations:** 1 College of Horticulture, Sichuan Agricultural University, Chengdu 611130, China; limy@sicau.edu.cn (M.L.); libinghua@stu.sicau.edu.cn (B.L.); 2019205007@stu.sicau.edu.cn (M.Y.); 2020205007@stu.sicau.edu.cn (L.W.); 2021205002@stu.sicau.edu.cn (G.H.); linyx@sicau.edu.cn (Y.L.); asyunting@sicau.edu.cn (Y.Z.); zhyong@sicau.edu.cn (Y.Z.); supnovel@sicau.edu.cn (Q.C.); wangyanwxy@sicau.edu.cn (Y.W.); hewen0724@gmail.com (W.H.); wangxr@sicau.edu.cn (X.W.); htang@sicau.edu.cn (H.T.); 2 Institute of Pomology & Olericulture, Sichuan Agricultural University, Chengdu 611130, China; 3 Departmental and Municipal Co-Construction of Crops Genetic Improvement of Hill Land Key Laboratory of Sichuan, Nanchong 637000, China

**Keywords:** cultivated strawberry, mitogen-activated protein kinases, genome-wide analysis, expression pattern, evolution, ABA and sucrose treatment, fruit ripening

## Abstract

Studies on many plants have shown that mitogen-activated protein kinases (MAPKs) are key proteins involved in regulating plant responses to biotic and abiotic stresses. However, their involvement in cultivated strawberry development and ripening remains unclear. In this study, 43 FaMAPK gene family members were identified in the genome of cultivated strawberry (*Fragaria* × *ananassa*), phylogenetic analysis indicated that FaMAPKs could be classified into four groups. Systematic analysis of the conserved motif, exon–intron structure showed that there were significant varieties between different groups in structure, but in the same group they were similar. Multiple *cis*-regulatory elements associated with phytohormone response, and abiotic and biotic stresses were predicted in the promoter regions of FaMAPK genes. Transcriptional analysis showed that all FaMAPK genes were expressed at all developmental stages. Meanwhile, the effect of exogenous ABA and sucrose on the expression profile of FaMAPKs was investigated. Exogenous ABA, sucrose, and ABA plus sucrose treatments upregulated the expression of FaMAPK genes and increased the content of endogenous ABA, sucrose, and anthocyanin in strawberry fruits, suggesting that ABA and sucrose might be involved in the FaMAPK-mediated regulation of strawberry fruit ripening. Based on the obtained results, MAPK genes closely related to the ripening of strawberries were screened to provide a theoretical basis and support for future research on strawberries.

## 1. Introduction

Plants encounter various biotic and abiotic stresses, such as drought, extreme temperatures, salinity and pathogens, throughout their life cycle. To adapt to these adverse stresses, plants have evolved to develop complex resistance mechanisms to maintain their integral structure and functions [1,2]. These resistance mechanisms rely on complex regulatory systems, including wide arrays of signal transduction pathways. Phosphorylation through protein kinases is among the main mechanisms in response to extracellular signaling and has been shown to have essential roles in plants’ stress resistance, growth, and development [3,4,5].

The mitogen-activated protein kinases (MAPKs) cascade is among the key pathways in eukaryotic signal transduction that modulate various cellular signaling cascades and influence the fundamental biological processes of eukaryotes [6,7]. The MAPK signaling cascade mainly comprises of three functional interlinked protein kinases: MAPK, MAPK kinase (MAPKK), and MAPKK kinase (MAPKKK). They operate as sequential signal transducers that transfer information from the cellular environment to transcriptional and metabolic response centers via phosphorylation. MAPKKKs are located upstream of multiple signal transduction cascades. They activate MAPKKs by phosphorylating the serine/threonine residues in the S/TXXXXXS/T (X represents any amino acid) motif, which sequentially phosphorylate the conserved threonine and tyrosine residues in the TXY motif of MAPKs to activate it. Activated MAPKs then act on different effector proteins in the cytoplasm or nucleus to regulate the expression of specific genes that enable plants to respond to biotic and abiotic stresses [8,9,10].

MAPKs comprise a family of serine/threonine protein kinases that contains 11 conserved domains. Phosphorylation of its activation TXY site occurs between VII and VIII subdomains. Plant MAPKs can be divided into four groups, A, B, C and D, based on TXY motifs. Groups A, B and C possess a TEY phosphorylation motif, while group D contains a TDY phosphorylation motif [8,11]. Studies have identified multiple MAPK genes in plants. For example, plant genome sequencing revealed 20 MAPKs in Arabidopsis [8], 19 in maize [12], 15 in rice [13], and 26 in apple [14]. Currently, a large number of functional studies on MAPK genes have been reported that they are involved in almost all activities of plants during their life cycle. For example, *AtMAPK4* and *AtMAPK6* in Arabidopsis were identified as important regulator genes of corresponding pathogen infection and abiotic stress in plants [15]. *AtMAPK9* and *AtMAPK12* are involved in biotic stress responses [16].

Abscisic acid (ABA) and sucrose have been shown to be among the central signaling molecules coordinating the ripening of fruits such as strawberry [17], tomato [18] and citrus [19]. It was also found that the regulation of fruit ripening by ABA is associated with sucrose [20]. As an important phytohormone, ABA participates in various signal transduction pathways, including MAPK signaling. It was shown that ABA activates MPK1 and MPK2 in an SRK2D/E/I-dependent manner and establishes a direct link between ABA and MAPK modules [21]. In Arabidopsis, the MAP3K17/18-MKK3-MPK1/2/7/14 cascade is induced by activating the ABA core signaling module following ABA treatment or drought-related stresses [22]. However, despite studies showing MAPK cascades as important signal transduction pathways in the downstream signals regulated by ABA, these have been limited to abiotic stresses.

Strawberry serves as an ideal model plant for studying non-climacteric fruit ripening. Although studies have shown that MAPKs are key proteins implicated in plant stress responses and ABA functions as one of the key signals regulating strawberries’ development, little is known about this gene family or their functions in cultivated strawberries (*Fragaria* × *ananassa*). In this study, we aimed to perform a comprehensive study on the gene structure, phylogeny, *cis*-acting element, evolutionary, and the expression level of the MAPK gene family in cultivated strawberries to provide a theoretical basis for improving cultivated strawberry’s stress resistance and development.

## 2. Results

### 2.1. Identification and Analysis of FaMAPKs in Cultivated Strawberry

After homologous alignment and conservative domain verification through the GDR database, a total of 43 genes encoding FaMAPK proteins were identified and renamed based on the homology of FvMAPKs (Table S1). The corresponding specific gene name, amino acid length, open reading frame (ORF) size, protein molecular weight, theoretical isoelectric point (*pI*), instability index, grand average of hydropathicity (GRAVY) and subcellular localizations are summarized in Table 1. The deduced length of FaMAPKs proteins ranged from 364 to 1010 amino acids. The amino acids are composed mainly of leucine (Leu), isoleucine (Ile) and glutamate (Glu). The ORF size of FaMAPKs ranged from 2099 bp (*FaMAPK7-4*) to 10,382 bp (*FaMAPK9-1*). In addition, the molecular weight of the 43 proteins ranged from 41.73 to 115.25 kD and the *pI* ranged from 5.05 to 9.34. The instability index ranged from 30.22 to 51.00. The GRAVY values of all proteins ranged from −0.773 to −0.184, suggesting that FaMAPKs are hydrophilic proteins [23]. Subcellular localization prediction revealed that most FaMAPK genes were located in the cytoplasm, while only a few were located in the nucleus, cytoskeleton, chloroplast, mitochondria and extracellular matrix.

**Figure 1:**
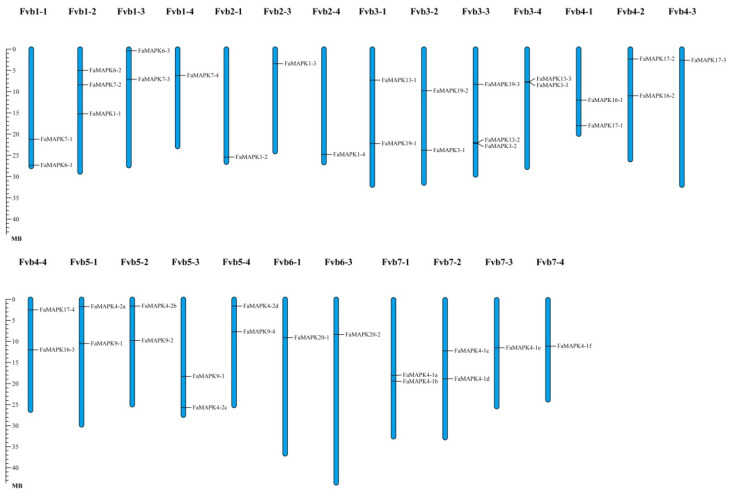
Chromosomal localization of the FaMAPKs. The chromosome number is indicated at the top of each chromosomal. The Fvb2-2, Fvb6-2 and Fvb6-4 chromosomes without FaMAPK gene localization are not showed.

These 43 FaMAPK genes were widely distributed in 25 of 28 cultivated strawberry chromosomes, whose gene numbers ranged from 1 to 3 (Figure 1). Fvb1-2 and Fvb3-3 had the highest number of FaMAPK genes, with three FaMAPK genes. In addition, the homologous genes were almost all located on adjacent chromosomes (except *FaMAPK1-3*). For example, *FaMAPK7-1*, *FaMAPK7-2*, *FaMAPK7-3*, and *FaMAPK7-4* were located on Fvb1-1, Fvb1-2, Fvb1-3, and Fvb1-4, respectively.

### 2.2. Phylogenetic Relationships and Multiple Sequences Alignment of FaMAPK Proteins

**Figure 2:**
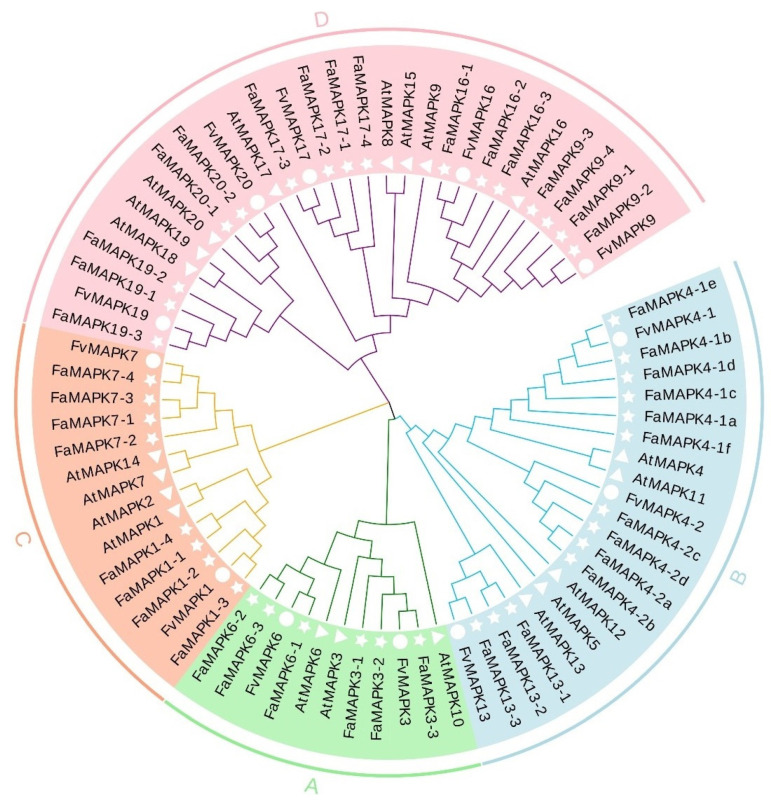
Phylogenetic tree of MAPK genes from Aradiopsis, *F. vesca* and *F. × ananassa* based on the amino acid sequences. Genes are grouped by different color blocks. The white triangles, circles and pentagrams indicated the MAPK genes in *Arabidopsis thaliana* (At); *F. vesca* (Fv); *F. × ananassa* (Fa).

To analyze the phylogenetic relationships and classification of the FaMAPK gene family members, a phylogenetic tree was constructed based on the amino acid sequences of 20 AtMAPKs from Arabidopsis, 12 FvMAPKs from *Fragaria vesca* and 43 FaMAPKs from *F. × ananassa* (Figure 2). We observed that all MAPK proteins were divided into four cluster groups: A, B, C, and D, which included 6, 13, 8, and 16 FaMAPKs, respectively.

To further analyze the sequence features of these 43 FaMAPK proteins, multiple sequence alignments of MAPK amino acid sequences were performed (Figures S1 and S2). The results showed a specific conserved motif on the N-terminal of the FaMAPKs. The conserved motifs of group B were RKY, SKY and DKY, while they were NKY and AKY in group A, TKY and SKY in group C, and NRY, SRY and SQY in group D. The conservative N-terminal motif was followed by a highly conservative region consisting of 11 featured domains. The FaMAPK proteins also contained P-loop, C-loop, Activation-loop and highly conserved distinct subdomains. The activate-loop subdomain, also known as T-loop, was located between the VII and VIII subdomains. All the FaMAPK family members contained a TXY motif. The conservative C-terminal motif also varied based on the groups of FaMAPKs. The core sequence was SDE in group B, SDY in group A, DNY in group C, and SKY (except FaMAPK19-1) in group D. In addition, the conserved domain in the (LH)DXXDE(P)XC (X represents any amino acid) sequence was detected only in groups A and B (except for FaMAPK13-1 and FaMAPK13-2), and is considered to act as a binding site for upstream MAPKK in the MAPK cascade. Multiple sequences alignment revealed that at least 58 gene pairs with more than 90% sequence similarity were homologous genes, and all pairs were located on their adjacent chromosome (Table S2). The highest percentage of amino acid sequence identity was found in FaMAPK4-1b/c, FaMAPK4-1b/d, FaMAPK4-1b/e, FaMAPK4-1c/d, FaMAPK4-1c/e and FaMAPK4-1d/e (100.00%).

### 2.3. Structure and Motif Location Analysis of FaMAPKs

**Figure 3:**
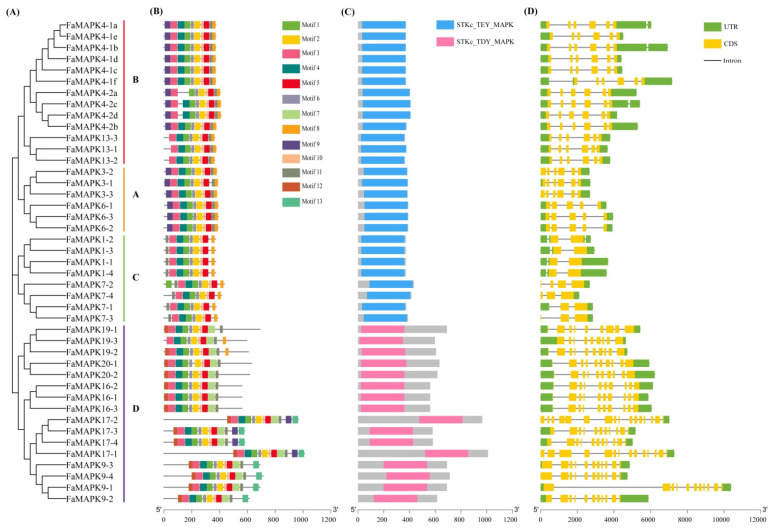
Phylogenetic relationships, conserved protein motifs and gene structure analysis of FaMAPK genes. (**A**) Phylogenetic tree of FaMAPK gene family. (**B**) Distribution of motifs of FaMAPK proteins. (**C**) The conserved domains in FaMAPK proteins. (**D**) Exon–intron structure of FaMAPK genes.

The gene structure of FaMAPKs was investigated to explore the various biological functions of the conserved domains (Figure 3). A total of 13 motifs were found in the FaMAPK sequences (Figure 3B and Table S3). Motifs 1–6 appeared in almost all FaMAPK members with the same number and distribution location, except for FaMAPK4-2a, which lacked motif 4, indicating that they are highly conservative. In addition, we observed that motifs 1–5 were annotated as protein kinase domains, indicating that all FaMAPKs belonged to the typical MAPK family.

MAPKs are a group of serine/threonine protein kinases widely studied in plants, of which the conserved threonine and tyrosine residues TXY represent an important ATP phosphate activation site. Based on their TXY motif, plant MAPKs can be classified into TEY and TDY subtypes, whereby X can represent E or D. The TDY motif is unique to plants. In this study, conserved domain analysis of the FaMAPK members showed that groups A, B and C had the same conserved domains (STKc_TEY_MAPK), while group D had the STKc_TDY_MAPK domain (Figure 3C). The exon–intron structure of the 43 FaMAPK genes was analyzed, and the results showed that the numbers of exons and introns of the same group were comparatively conserved (Figure 3D). In contrast, the numbers of exons and introns in group D were significantly more extended than other groups, suggesting that the function of group D might be different from the other three groups.

### 2.4. Synteny Analysis of the MAPK Genes in Arabidopsis, F. vesca and F. × Ananassa

**Figure 4:**
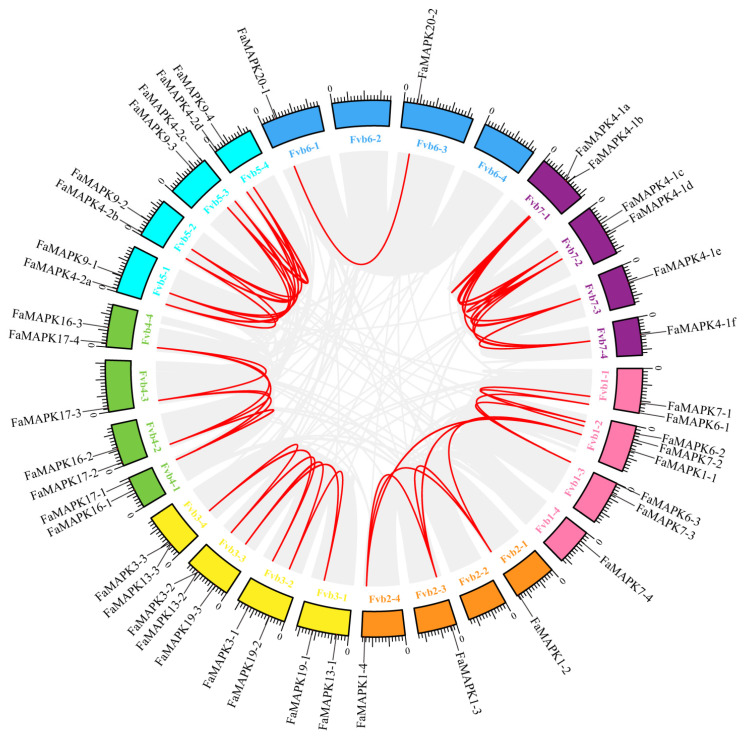
Segmental duplication and synteny analysis of FaMAPKs. Chromosome of cultivated strawberry was distinguished by different color. Each FaMAPK gene is marked with a short line on the chromosome, and the red curve indicates a collinear gene pair.

**Figure 5:**
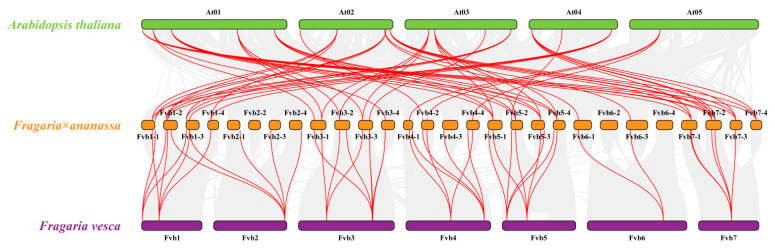
Collinearity analysis of MAPK genes among Arabidopsis, *F. × ananassa* and *F. vesca* genomes. Colored circular rectangles denote the chromosomes of three plants. Grey curves indicate collinear blocks within the genomes, and the red curves represent gene pairs that are collinear with MAPK genes.

Collinearity analysis identified that 49 FaMAPK gene pairs in *F. × ananassa* were collinear pairs (Figure 4). Most of the FaMAPKs pairs co-located in the same or adjacent chromosome were fragmental duplications. To determine the evolutionary relationship and degree of homology among Arabidopsis, *F. vesca* and *F. × ananassa*, their comparative synteny at the genome level was analyzed to elucidate the FaMAPKs’ origin (Figure 5). We observed 41,047 synteny blocks between Arabidopsis and *F.* × *ananassa* genomes, of which 30.24% were collinear. Comparatively, there were 866,968 synteny blocks between *F. vesca* and *F. × ananassa* genomes. Collinearity analysis of FaMAPKs and FvMAPKs showed that *F. × ananassa* has a close relative of *F. vesca*, and that there could have been a four-time relationship for doubling and replicating the chromosomes and genes between them during evolution.

### 2.5. Analysis of Cis-Regulatory Elements of FaMAPK Genes

**Figure 6:**
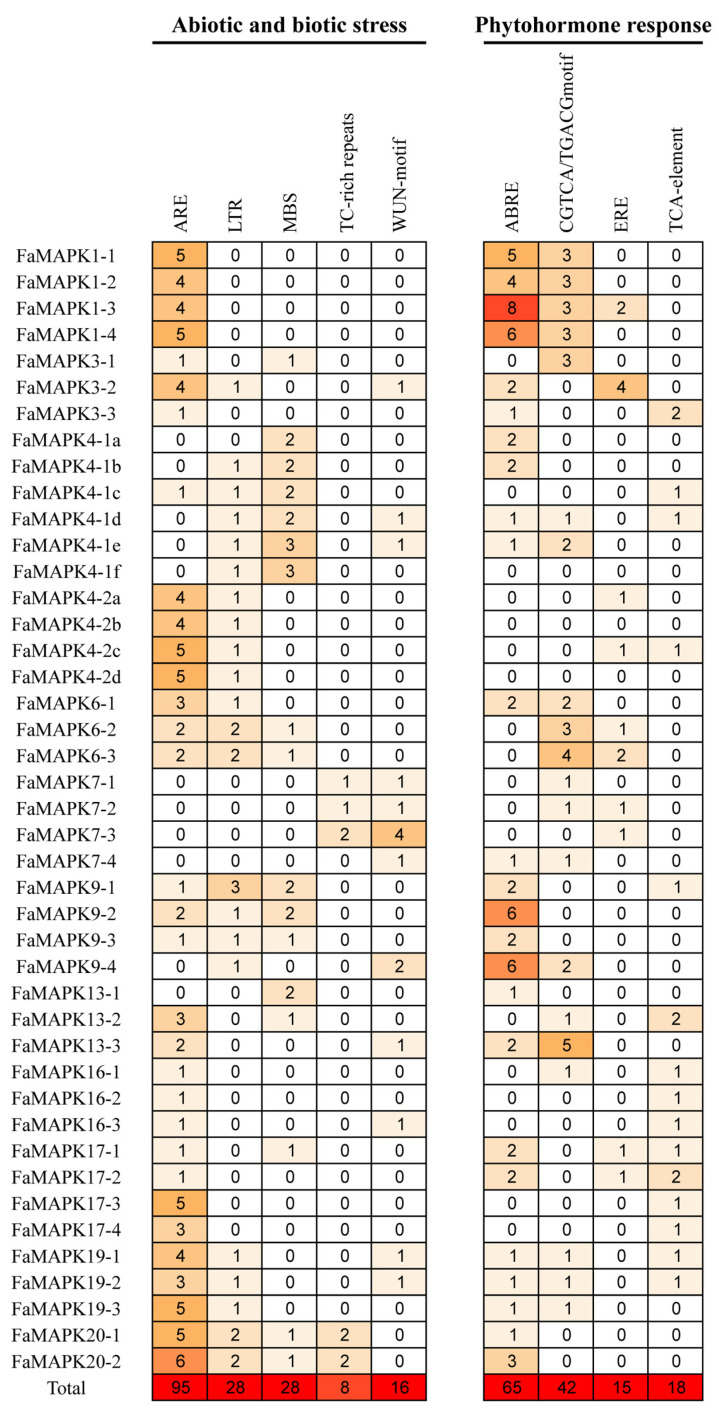
Analysis of *cis*-regulatory elements in the promoter regions of FaMAPK genes. The figures indicate the number of each *cis*-regulatory element in the promoter region (1.5 kb upstream of the translation start site) of the FaMAPK genes. Based on functional annotations, the *cis*-regulatory elements can be classified into two major classes: phytohormone response-related, and abiotic and biotic stresses-related *cis*-elements.

All *cis*-regulatory elements in the promoter regions of FaMAPK genes were analyzed. They were classified into two groups based on their functional associations: abiotic and biotic stresses (ARE, LTR, MBS, TC-rich repeats and WUN-motif), and phytohormone responses (ABRE, CGTCA motif/TGACG motif, ERE and TCA-element) (Figure 6 and Figure S3). Except for *FaMAPK4-1a*/*b*/*d*/*e*/*f*, *FaMAPK7-1*/*2*/*3*/*4*, *FaMAPK9-4* and *FaMAPK13-1*, all the other FaMAPK genes contained the antioxidant response element (ARE), which represents the *cis*-acting regulatory element essential for anaerobic induction. The low-temperature responsiveness element (LRT), drought inducibility element (MBS), defense and stress responsiveness element (TC-rich repeats), and wound responsive element (WUN-motif) appeared 28, 28, 8, and 16 times, respectively. The abscisic acid responsiveness (ABRE), MeJA responsive element (CGTCA motif/TGACG motif), ethylene response element (ERE), and salicylic acid response element (TCA-element) appeared 65, 42, 15, and 18 times, respectively. Interestingly, most FaMAPK gene promoters, except for *FaMAPK4-1f*, *FaMAPK4-2b* and *FaMAPK4-2d*, contained one or more phytohormone response elements. These results suggest that the FaMAPK gene family may have important roles in the physiological functions of plants.

### 2.6. Transcript Abundance Analysis of FaMAPK Genes in Strawberry

**Figure 7:**
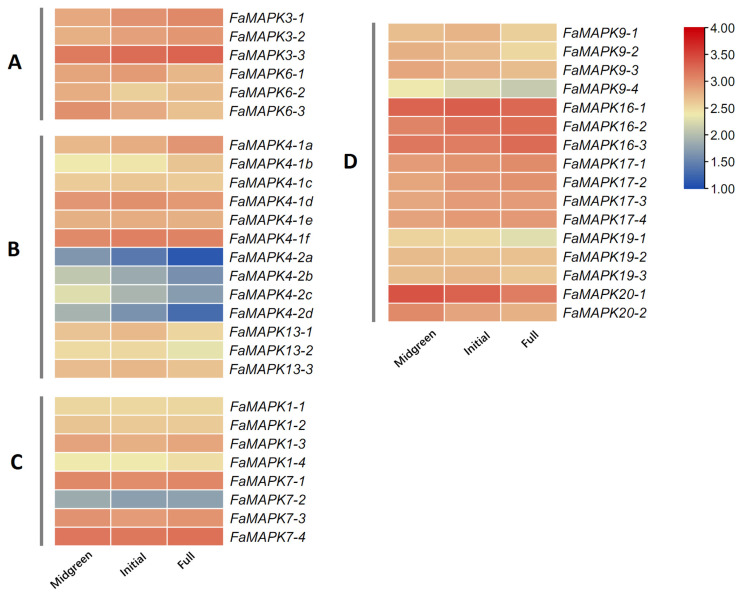
Transcript abundance of FaMAPK genes at different fruit developmental stages. FPKM values of FaMAPK genes were transformed by log2(FPKM + 1), and the heatmap was constructed with TBtools software. Midgreen: medium green stage; Initial: initial red stage; Full: full-red stage.

To investigate the role of MAPK genes during fruit development, we examined the developmental expression pattern of the FaMAPK genes. We observed that all FaMAPK genes were expressed in the developmental stages (Figure 7). Most of the FaMAPK genes were highly expressed, especially in groups A and D. *FaMAPK3-3*, *7-4*, *16-1*, and *20-1* demonstrated the highest expression, while *FaMAPK4-2a*, *4-2b*, *4-2c*, and *4-2d* demonstrated the weakest expression. The gene expressions of homologous gene pairs were similar.

**Figure 8:**
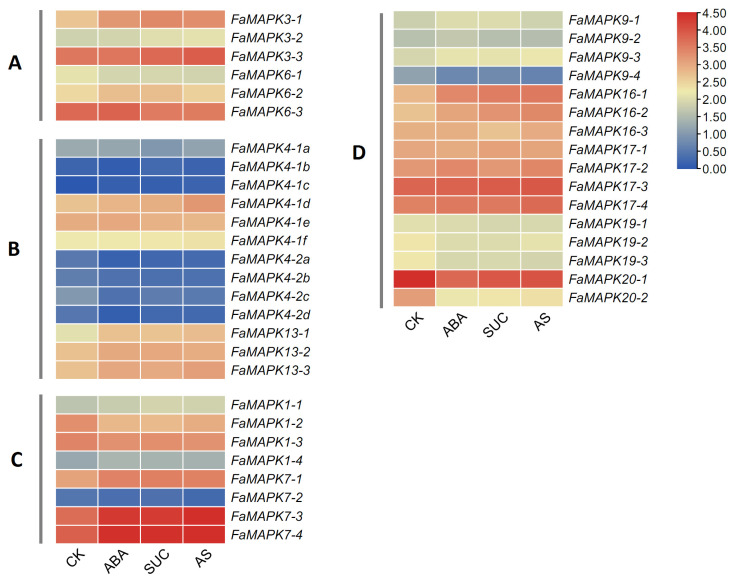
Transcript abundance of FaMAPK genes at exogenous ABA and sucrose treatments. CK: control; ABA: abscisic acid; SUC: sucrose; AS: the mixture of abscisic acid and sucrose. FPKM values of FaMAPK genes were transformed by log2(FPKM + 1), and the heatmap was constructed with TBtools software.

To investigate the underlying regulatory mechanism of MAPK genes during fruit ripening, we examined the expression pattern of the FaMAPK genes in response to exogenous ABA and sucrose. Our findings showed differential expression patterns of FaMAPK genes after exogenous ABA and sucrose treatments (Figure 8). The expression levels within homologous genes varied significantly. For instance, the expression of *FaMAPK3-1* and *3-3* was upregulated in response to treatments, while that of *FaMAPK3-2* did not change. Further, we observed that ABA and ABA plus sucrose induced the expression of several MAPK genes, such as *FaMAPK3-1*, *3-3*, *7-3*, and *7-4*. In contrast, the expression of FaMAPK genes in group B was relatively low, while *FaMAPK20-1* was highest in CK but decreased following treatments in strawberry fruit.

### 2.7. Validation of the Expression of FaMAPK Genes by qRT-PCR

**Figure 9:**
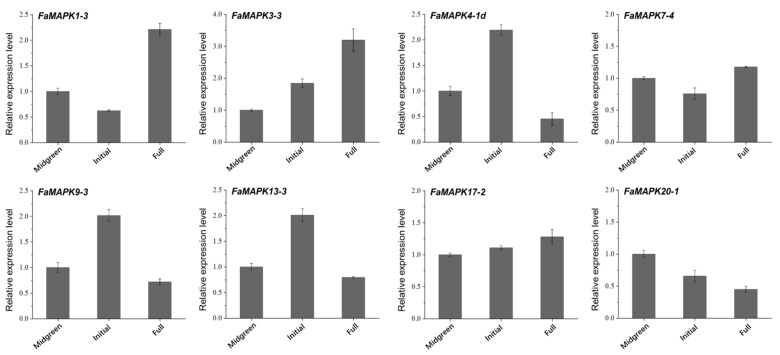
Expression profiles of FaMAPK genes at different fruit developmental stages.

qRT-PCR analysis was performed to validate the accuracy of the transcriptome profiles. We quantified the expression of eight FaMAPK genes during fruit development and further explored the impact of exogenous ABA and sucrose on gene expression during fruit ripening. Our findings showed that these eight FaMAPK genes demonstrated stage-specific expression patterns during fruit development (Figure 9). The expression levels of *FaMAPK1-3* and *3-3* peaked in the full-red stage, while that of *FaMAPK 4-1d*, *9-3* and *13-3* increased significantly and peaked in the initial red stage. The expression of *FaMAPK20-1* decreased in the developmental stage. No significant change was detected in the expression of *FaMAPK7-4* and *17-2*.

**Figure 10:**
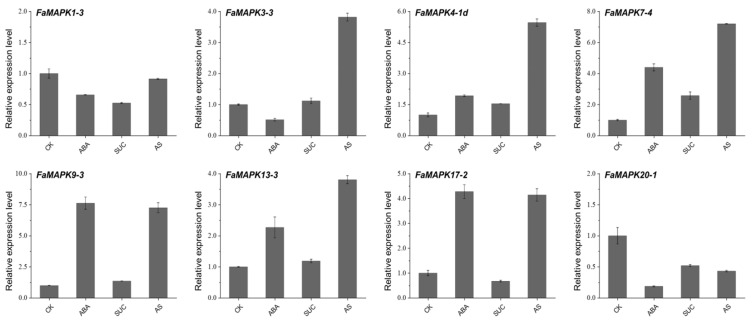
Expression profiles of FaMAPK genes at exogenous ABA and sucrose treatments.

After ABA treatment, the expression of four genes, including *FaMAPK7-4*, *9-3*, *13-3* and *17-2*, was significantly upregulated compared with control, whereas that of *FaMAPK4-1d* and *7-4* increased under the sucrose treatment (Figure 10). In addition, the relative expression of six genes, except for *FaMAPK1-3* and *20-1*, was highly expressed under the ABA plus sucrose treatment. The expression of *FaMAPK 20-1*, only under ABA treatment, was decreased. Overall, the results of RNA-Seq and qRT-PCR analysis were consistent, suggesting that the FaMAPK genes may be involved in the developmental and ripening processes of cultivated strawberries.

### 2.8. Effects of Exogenous ABA and Sucrose on Strawberry Fruit Ripening

**Figure 11:**
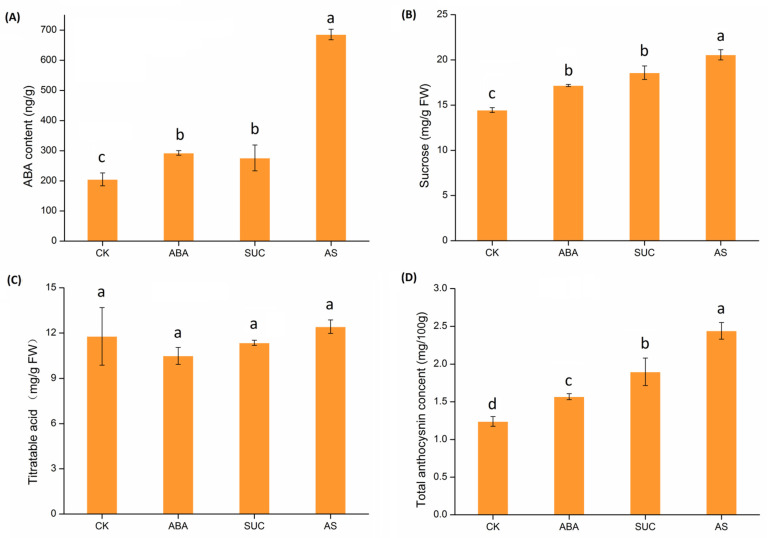
Effect of exogenous sucrose and ABA on the development of strawberry fruits. (**A**) ABA content; (**B**) Sucrose content; (**C**) Titratable acid content; (**D**) Total anthocyanin content. CK: control; ABA: abscisic acid; SUC: sucrose; AS: the mixture of abscisic acid and sucrose. Each bar represents the mean ± SD, *T*-test at the significance level of 0.05 (*p* < 0.05), different letters (a–d) indicate significant difference, and the same letters indicate insignificant differences.

The ABA, sucrose and total anthocyanin content in strawberry fruits varied with different treatments, and the difference in the content of titratable acid among these treatments was not significant (Figure 11). The highest ABA content in the fruits was found in the ABA plus sucrose treatment, followed by ABA-only and sucrose-only treatments. The ABA content in all treatment groups was all significantly higher than in the control group. The sucrose content demonstrated the same variation trends as ABA content, with the sucrose content under ABA plus sucrose treatment being higher than that under ABA or sucrose treatments. Compared with control, the total anthocyanin content increased significantly in response to ABA, sucrose, and ABA plus sucrose. The difference in total anthocyanin content between the three treatments was highly significant.

Correlation analysis showed that ABA, sucrose, acid, and anthocyanin changes were positively correlated, implying that ABA and sucrose had a role in strawberry ripening (Table 2). The expression of most FaMAPK genes was significantly and positively correlated with the changes of ABA, sucrose, titratable acid and total anthocyanin. The content of ABA was highly correlated with the relative expression *FaMAPK3-3* and *FaMAPK4-1d*, indicating that these genes could promote the ripening of strawberry fruits. In contrast, the expression of *FaMAPK20-1* was negatively correlated with ABA, sucrose and anthocyanin. Overall, the trend of gene expression was the same as that of the accumulation of ABA, sucrose and anthocyanin, suggesting that sucrose and ABA could co-regulate fruit ripening by influencing the expressions of FaMAPK genes.

## 3. Discussion

MAPKs belong to the serine/threonine-protein kinase family. At present, the functions of MAPK genes have been well studied in Arabidopsis, rice, maize and other plants. However, there is little knowledge on the role of the MAPK gene family in cultivated strawberry, which is one of the most popular fruits, well known for its nutritional value and unique flavor. In this study, we identified a total of 43 MAPK genes in cultivated strawberries, which is greater than the number of identified MAPK genes in any other plant species, such as Arabidopsis (20 genes) [8], rice (15 genes) [13], wild strawberry (12 genes) [24], barley (20 genes) [25] and *Fagopyrum tataricum* (16 genes) [26]. Multiple sequences alignment and phylogenetic tree analysis showed that the FaMAPK family members could be classified into four groups based on TXY motifs. Groups A, B and C contained a TEY amino acid motif, while group D contained a TDY amino acid motif. A close genetic association was found between cultivated and wild strawberries in terms of MAPK genes and demonstrated consistent subgroups division [24], indicating that MAPK members in strawberries are relatively conservative in evolution. During the long evolution of plants, many genes have either expanded or contracted. There are reports showing a number of plant species that retained some characteristics of their common ancestors and also developed their own unique characteristics. However, the gene number of each group varies within different species, and group D was demonstrated to have the largest number of members. Here, we found that the number of group B members in cultivated strawberries was significantly greater than other species, suggesting that this group had undergone significant expansion through the evolution of cultivated strawberries. These may have contributed to the ability of strawberries to adapt to significant environmental changes and retain a key role in plant adaptation.

The gene structure of a family member is closely related to its gene expression and functional variations [27]. All members of the FaMAPK gene family contain motifs 1–5, which are the conserved motifs of the MAPK family. Additionally, there are 1–2 unique motifs between different groups, indicating that the conserved motifs in the MAPK genes support their close evolutionary relationship, while there might be different functional divisions when participating in biological processes between different groups [7]. The distribution of introns and exons in the gene structure of the MAPK family in cultivated strawberries also demonstrated some extent of regularity, for instance, group C contains only 3–4 exons, while group D contains more than 10 exons. Similar intron/exon structural patterns of MAPKs were observed in other plants, representing the highest level of intron/exon variations between groups and high conservation within a group. *Cis*-acting elements, such as ABA-responsive elements (ABRE), methyl jasmonate (MeJA)-responsive motifs (CGTCA motif and TGACG motif), low temperature-responsive (LTR) and antioxidant response element (ARE), play key roles in plant growth, development and stress responses [28,29]. In this study, we found that the promoter of FaMAPK genes was enriched with many *cis*-elements, namely ARE, ABRE and MeJA response elements, suggesting that they may have crucial roles in the regulation of different plant processes such as growth, development and stress responses. The promoters of FaMAPK genes are enriched with a large number of *cis*-elements, especially ARE and ABRE and methyl jasmonate-responsive element (CGGTCA-motif), which are speculated to be involved in growth and development and various biological processes. Previous studies have also found that MeJA and ABA response elements were abundant in the promoters of MAPK genes of cotton [30] and kiwifruits [31].

Currently, there is substantial evidence showing that MAPK genes are implicated in regulating almost all life activities of plants, including plant growth and immune response. *AtMAPK3* and *AtMAPK6* were initially thought to be involved in stress responses and hormone signal transduction but were later found to be also involved in plant growth and development [32]. Deletion of *mapk4* was shown to result in a mutant defect during meiotic cytokinesis [33]. Another study reported that the OsMKKK10-OsMKK4-OsMAPK6 cascade positively regulates grain size and weight in rice [34]. However, there are limited reports on the involvement of the MAPK cascades in the development and maturation of strawberries. In this study, the expression patterns of the MAPK family members during strawberry fruit development were analyzed. We observed that all MAPKs were expressed in the fruit’s developmental stages, and the expression levels of groups A and D were high, indicating that these two groups have important roles in the development of strawberries at the morphological level. Promoter analysis of the members of these two groups revealed that they contained multiple *cis*-elements, for instance, ABRE and CGTCA-motif, that are related to plant development and hormonal regulation.

ABA and sucrose was shown to have key roles in the regulation of fruit development [35]. To further explore the impact of exogenous ABA and sucrose on strawberry fruit ripening and gene regulation, different treatments of exogenous ABA, sucrose, and ABA plus sucrose were used to spray on fruits. Compared with control, the three treatments used could promote the content of endogenous ABA, sugar and anthocyanin in mature strawberries, while fruits that were treated with ABA plus sucrose had the highest content. External application of sucrose and ABA promoted the rapid accumulation of anthocyanins, which was confirmed in bilberry [36], grape [37] and strawberry [38]. Further, the external application of sucrose was shown to promote the increase of endogenous ABA content in strawberry fruits [20,39]. Therefore, it is speculated that the external application of sucrose could indirectly promote anthocyanin accumulation and fruit maturation by inducing an increase in endogenous ABA. Previous studies identified a signal connection between ABA and MAPK modules, such as OsMAPK5 in rice [40] and MdMPK1 in apple [41], which could also be activated by ABA. In this study, based on transcriptome expression abundance and qRT-PCR validation, we found that ABA could significantly promote the expression of several MAPK genes. In addition, exogenous ABA and ABA plus sucrose could also promote the expression of MAPK genes, especially *FaMAPK3-1*, *3-3*, *7-3* and *7-4*. Here, we showed that treatment with ABA plus sucrose demonstrated the strongest induction effect on the expression of MAPK genes and had the most significant effect in promoting fruit ripening and anthocyanin accumulation, indicating a synergistic or superposition effect between them.

## 4. Materials and Methods

### 4.1. Characterization, Phylogenetic, and Physicochemical Properties Analysis of MAPK Genes in the F. × ananassa Genome

The *F. × ananassa* genome sequence data used in our study are available within the GDR database (https://www.rosaceae.org/; accessed on 1 July 2021). Using local blast-p with BioEdit software, the 20 amino acid sequences of MAPK genes in Arabidopsis and the 12 in wild strawberry were used as query sequences to identify homologous genes. All the putative MAPK proteins were screened using Pfam (http://pfam.xfam.org/; accessed on 1 July 2021), NCBI CDD (https://www.ncbi.nlm.nih.gov/cdd/; accessed on 1 July 2021) and InterProScan (http://www.ebi.ac.uk/interpro/; accessed on 1 July 2021) to remove the typical conservative sequences of Serine/Threonine protein kinases and the most fundamental TDY or TEY markers in the activation loop motif. All the non-redundant gene sequences encoding complete amino acid sequences were considered as FaMAPK genes.

Using the ProtParam tool (http://www.expasy.org/tools/protparam.html; accessed on 5 July 2021), amino acid numbers, molecular weights, predicted theoretical isoelectric points (pI), instability index and chemical properties data were predicted. Using ClustalX software and setting default parameters, amino acid sequences were performed by multiple alignment, while GeneDoc software was used to modify manually. Accounting to previous studies of MAPK proteins in Arabidopsis, apple and wild strawberry, MEGA was used to construct a phylogenetic tree based on the neighbor joining (N–J) method with a bootstrap value of 1000. The tree was beautified by using the Evolview website (http://120.202.110.254:8220/evolview; accessed on 1 July 2021). The specific conserved domains in the MAPK family were searched on NCBI (https://www.ncbi.nlm.nih.gov; accessed on 1 July 2021) and the conserved domains were predicted by InterProScan (http://www.ebi.ac.uk/Tools/InterProScan/; accessed on 1 July 2021). MEME was used to identify conserved motifs and TBtools software was used to visualize the evolution motifs and e genetic structures of the MAPK family in cultivated strawberry. Subcellular localization analysis was performed Using WOLF PSORT (https://www.genscript.com/wolf-psort.html/; accessed on 5 July 2021). All *cis*-regulatory elements in the 1500 bp upstream gene promoter are available through the PlantCARE website (http://bioinformatics.psb.ugent.be/webtools/plantcare/html/; accessed on 20 July 2021). The homologous gene pair of the MAPK gene family in *F.* × *ananassa*, *F. vesca* and Arabidopsis were identified using the OrthoMCL software (https://orthomcl.org/orthomcl/; accessed on 20 July 2021). Gene pair collinearity was determined using the MCScanX software (http://chibba.pgml.uga.edu/mcscan2; accessed on 20 July 2021) and then plotted using the Circos software (http://circos.ca/software/download/; accessed on 20 July 2021).

### 4.2. Plant Materials and Treatments

The experimental samples were strawberry cultivar *F.* × *ananassa* cv. Bebihoppe, which were selected for listing at the flowering stage with consistent growth. The fruits at midgreen, initial red and full-red stages were collected separately. The concentrations of exogenous ABA and sucrose refer to the previous reports by our research group [39]. A total of 500 strawberry fruits from at least 100 plants were in similar shape and size and without physical injuries or microorganisms infection, and were randomly selected and tagged. With water as the control, the strawberry fruits at the de-greening stage (18 days after anthesis) were sprayed with 95 uM ABA, 100 mM sucrose and the mixture of ABA and sucrose (1:1) at the degreening stage (18 d after anthesis) until dripping. The fruits were harvested on the 8th day after spraying, and quickly frozen in liquid nitrogen and then stored at −80 °C for further analysis.

### 4.3. RNA Extraction and cDNA Synthesis

Accounting to the improved CTAB method [42], total RNA was extracted from fruits at different treatments with slight modifications. The extracted RNA was quantified by nucleic acid protein instrument and electrophoresed on a 1.0% agarose gel to verify its integrity. First-strand cDNA was synthesized referring to the instructions of the reverse transcription kit of Beijing Quanshijin Biotechnology Co., Ltd. (Beijing, China).

### 4.4. Quantitative Transcript Analysis and Quantitative Real-Time PCR (qRT-PCR) Verification

The transcript abundance of all MAPK genes in strawberry was obtained from the transcriptome sequencing data that had been submitted to a public database (NCBI: PRJNA565646, PRJNA552213). The transcript abundance of FaMAPK genes in different development stages and treatments was counted by HTSeq [43], and FPKM (fragments per kilo base of exon per million fragments mapped) was then calculated to estimate the expression level [44].

Among all genes, eight FaMAPKs that might be associated with fruit ripening were selected to examine their expression. qRT-PCR was performed with SYBR qPCR Mix (Vazyme Biotech Co., Nanjing, China) using a Bio-Rad CFX96 real-Time System (Bio-Rad, CA, USA). The experiments were repeated in three independent bio-replicates and tech-replicates, and *FaActin* was used as the internal control to normalize gene expression. The relative expression levels of FaMAPKs were calculated based on the 2^−ΔΔCT^ method. The primer sequences were designed using Primer Premier 5.0 software (version 5.0; Premier Biosoft International: Palo Alto, CA) and the sequence information is listed in Table S4.

### 4.5. Determination of ABA, Sucrose, Titratable Acid, and Total Anthocysnin Content

The fruit ABA content was measured by a plant ABA ELISA Kit (Jiancheng, Nanjing, China). The sucrose content was determined by the plant tissue sucrose content detection kit (Jiancheng, Nanjing, China). The titratable acid (total acid) content was measured by phenolphthalein titration. Total anthocyanins were measured by the method depicted [45]. Each treatment included three replicates, and each repeat included 10 fruit individuals.

## 5. Conclusions

In this study, a total of 43 MAPK gene family members were identified in *F. × ananassa* We performed a comprehensive analysis of the FaMAPK family genes, including phylogeny, chromosomal localization, gene structure, *cis*-regulatory element, evolution, and expression profiling. An in-depth study was performed to clarify the involvement of FaMAPK genes in fruit developing and ripening. The results of this study provide a theoretical basis for enriching MAPKs to regulate strawberry ripening and enhance our understanding of the molecular mechanism of key genes regulated by ABA and sucrose in strawberry ripening.

## Figures and Tables

**Table 1:** Physical and chemical aspects of MAPK genes in the cultivated strawberry.

Gene Name	Amino Acid/aa	ORF/bp	Molecular Weight/kD	*pI*	Instability Index	GRAVY	Subcellular Localization
FaMAPK1-1	375	3674	42.76	5.96	39.12	−0.265	Cytoplasm
FaMAPK1-2	372	2731	42.84	6.09	38.82	−0.277	Cytoplasm
FaMAPK1-3	372	2929	42.81	6.09	37.31	−0.282	Cytoplasm
FaMAPK1-4	372	3597	42.73	5.96	37.71	−0.272	Cytoplasm
FaMAPK3-1	388	2703	44.59	5.52	37.52	−0.196	Cytoplasm
FaMAPK3-2	371	3006	42.64	5.54	38.41	−0.251	Cytoplasm
FaMAPK3-3	371	2919	42.71	5.62	39.46	−0.266	Cytoplasm and Nucleus
FaMAPK4-1a	373	6027	42.72	6.08	44.34	−0.337	Cytoplasm
FaMAPK4-1b	373	6924	42.67	6.08	43.90	−0.329	Cytoplasm
FaMAPK4-1c	373	4449	42.67	6.08	43.90	−0.329	Cytoplasm
FaMAPK4-1d	373	4405	42.67	6.08	43.90	−0.329	Cytoplasm
FaMAPK4-1e	373	4499	42.67	6.08	43.90	−0.329	Cytoplasm
FaMAPK4-1f	373	7167	42.68	6.08	42.19	−0.329	Cytoplasm
FaMAPK4-2a	405	5228	45.92	6.17	45.52	−0.281	Cytoplasm
FaMAPK4-2b	377	5289	43.23	6.28	45.32	−0.368	Cytoplasm
FaMAPK4-2c	410	5413	46.65	6.54	45.05	−0.298	Cytoplasm
FaMAPK4-2d	410	4164	46.68	6.43	44.41	−0.301	Cytoplasm
FaMAPK6-1	391	3584	44.75	5.60	40.29	−0.292	Cytoskeleton
FaMAPK6-2	390	3913	44.71	5.46	41.08	−0.312	Nucleus
FaMAPK6-3	391	3948	44.77	5.58	39.98	−0.277	Cytoskeleton
FaMAPK7-1	376	2842	43.41	8.02	33.67	−0.219	Cytoplasm
FaMAPK7-2	434	2675	49.68	9.02	39.13	−0.366	Cytoplasm
FaMAPK7-3	389	2846	44.84	8.57	34.20	−0.205	Cytoplasm
FaMAPK7-4	415	2099	47.61	8.63	30.22	−0.246	Cytoplasm
FaMAPK9-1	691	10,382	78.41	8.07	44.61	−0.644	Chloroplast
FaMAPK9-2	615	5875	69.64	6.40	41.81	−0.592	Mitochondria
FaMAPK9-3	692	4853	78.07	6.39	41.57	−0.580	Nucleus
FaMAPK9-4	713	4733	80.47	6.76	41.94	−0.579	Cytoplasm
FaMAPK13-1	377	3648	43.06	5.16	44.44	−0.184	Cytoplasm
FaMAPK13-2	364	3789	41.73	5.16	43.42	−0.268	Extracellular matrix
FaMAPK13-3	365	3794	41.83	5.05	42.04	−0.284	Extracellular matrix
FaMAPK16-1	561	5872	63.80	8.73	36.05	−0.476	Cytoplasm
FaMAPK16-2	561	6121	63.77	8.64	35.21	−0.483	Cytoplasm
FaMAPK16-3	561	6052	63.82	8.80	36.10	−0.477	Cytoplasm
FaMAPK17-1	1010	7286	115.25	8.21	50.90	−0.770	Nucleus
FaMAPK17-2	966	7019	109.89	8.21	51.00	−0.773	Nucleus
FaMAPK17-3	580	5173	66.33	7.07	38.18	−0.548	Chloroplast
FaMAPK17-4	582	5018	66.35	7.34	39.18	−0.546	Chloroplast
FaMAPK19-1	691	5431	78.60	9.29	33.20	−0.246	Cytoplasm
FaMAPK19-2	607	4715	68.88	9.34	31.18	−0.392	Cytoplasm
FaMAPK19-3	597	4641	67.93	9.23	32.44	−0.414	Cytoplasm
FaMAPK20-1	633	5919	72.43	9.11	38.10	−0.446	Cytoplasm
FaMAPK20-2	618	6221	70.66	9.23	37.11	−0.484	Cytoplasm

**Table 2:** Correlation coefficient between relative expression level of FaMAPK genes with ABA, sucrose, titratable acid and total anthocyanin content under exogenous sucrose and abscisic acid treatments.

	Expression Level								ABA Content	Sucrose Content	Titratable Acid Content	Total Anthocysnin Content
	*FaMAPK1-3*	*FaMAPK3-3*	*FaMAPK4-1d*	*FaMAPK7-4*	*FaMAPK9-3*	*FaMAPK13-3*	*FaMAPK17-2*	*FaMAPK20-1*				
ABA content	0.280	0.955 *	0.999 **	0.923	0.632	0.943	0.628	−0.360	1			
Sucrose content	−0.012	0.864	0.789	0.646	0.170	0.606	0.128	−0.175	0.816	1		
Titratable acid content	0.651	0.838	0.615	0.298	−0.128	0.392	−0.092	0.470	0.641	0.707	1	
Total anthocysnin content	−0.099	0.855	0.893	0.853	0.482	0.798	0.434	−0.473	0.906	0.941	0.524	1

* means significant differences at 0.05 level; ** means significant differences at 0.01 level.

## Data Availability

The genome sequence data is available at GDR database (GDR, https://www.rosaceae.org (accessed on 1 July 2021)). The RNA-seq data were downloaded from SRA database of NCBI (Accession ID: PRJNA565646 and PRJNA552213; Link: https://www.ncbi.nlm.nih.gov/sra/PRJNA565646 (accessed on 1 July 2021) and https://www.ncbi.nlm.nih.gov/sra/PRJNA552213 (accessed on 1 July 2021)).

## Supplementary Materials

The following supporting information can be downloaded at: https://www.mdpi.com/article/10.3390/ijms23095201/s1.
