# Supplementary figures and images for: Genome-Wide Identification and Expression of MAPK Gene Family in Cultivated Strawberry and Their Involvement in Fruit Developing and Ripening
Source: Int J Mol Sci. 2022 May 6;23(9):5201. doi: 10.3390/ijms23095201 (PMC9104773; doi:10.3390/ijms23095201)

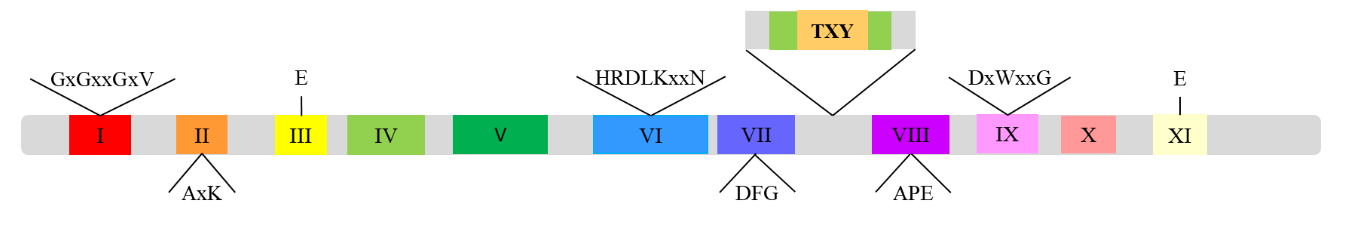

Supplement: Supplementary file 1 [file ijms-23-05201-s001.zip › Figure S1.png]

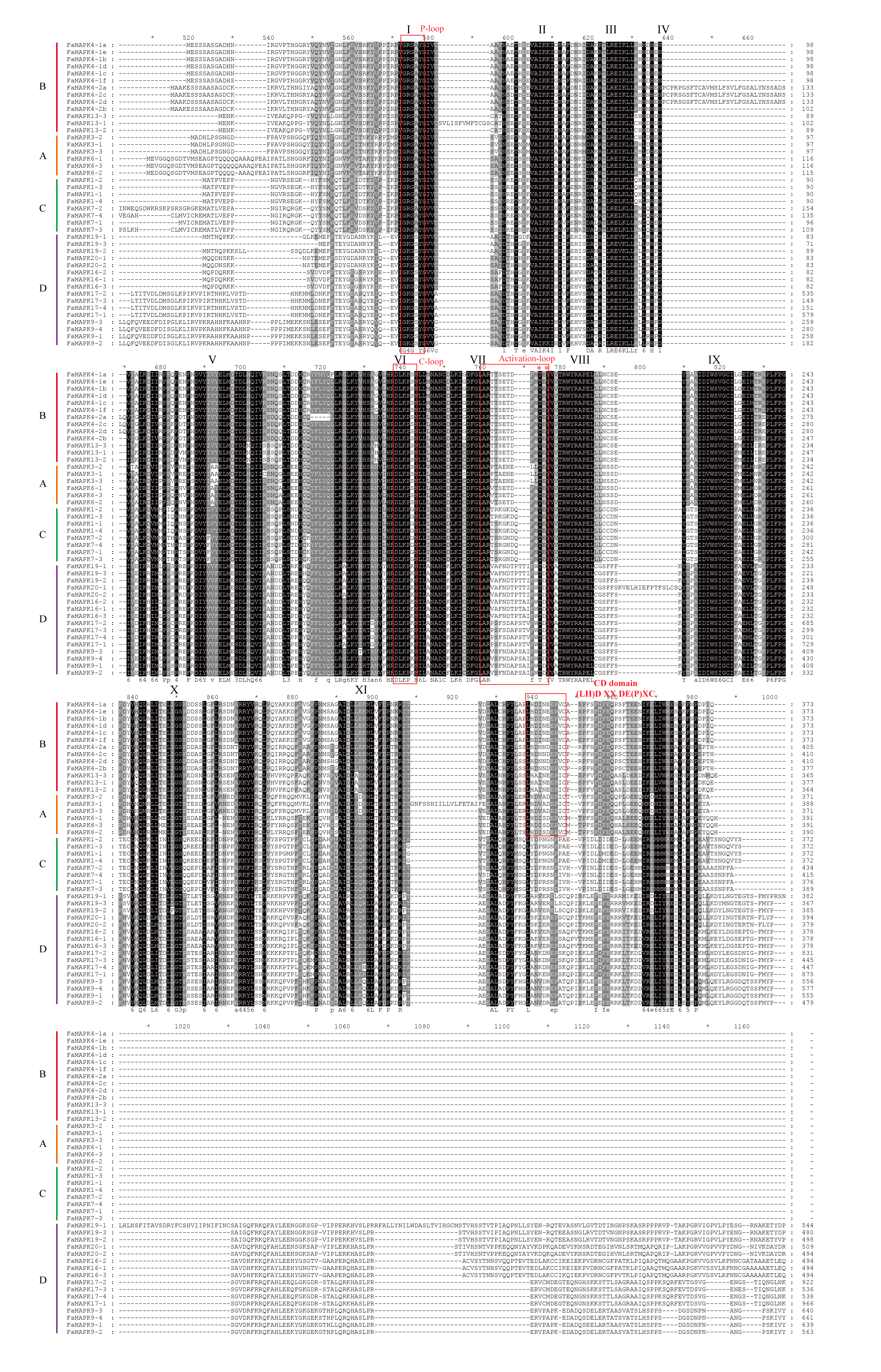

Supplement: Supplementary file 1 [file ijms-23-05201-s001.zip › Figure S2.png]

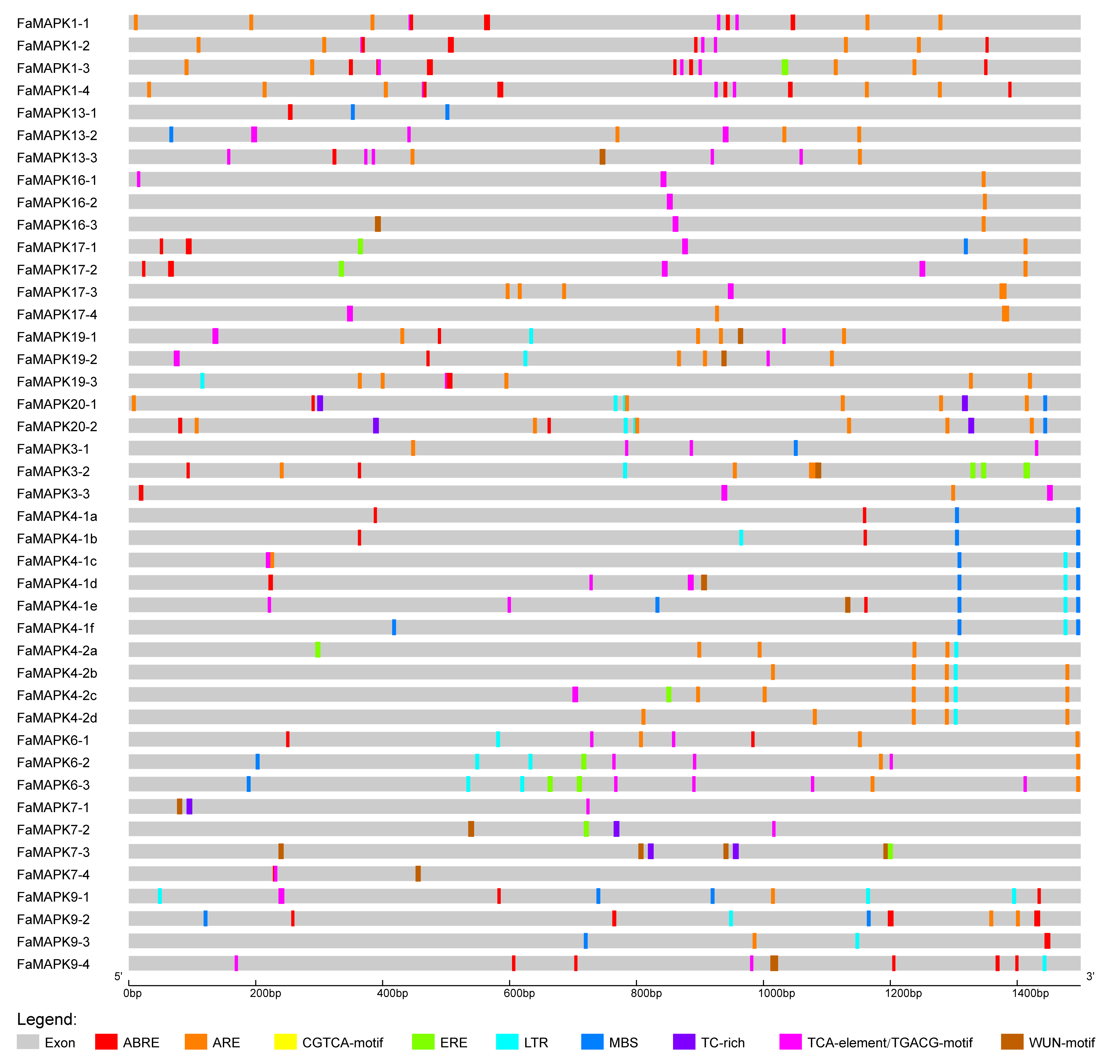

Supplement: Supplementary file 1 [file ijms-23-05201-s001.zip › Figure S3.png]
